# Supplementary material for: Seminoma and Embryonal Carcinoma Footprints Identified by Analysis of Integrated Genome-Wide Epigenetic and Expression Profiles of Germ Cell Cancer Cell Lines
Source: PLoS One. 2014 Jun 2;9(6):e98330. doi: 10.1371/journal.pone.0098330 (PMC4041891; doi:10.1371/journal.pone.0098330)
Supplement: File S1 — ZIP file containing DMRforPairs output for significant regions. Please start from the html files. (ZIP) [file pone.0098330.s008.zip › figures/31664.pdf]

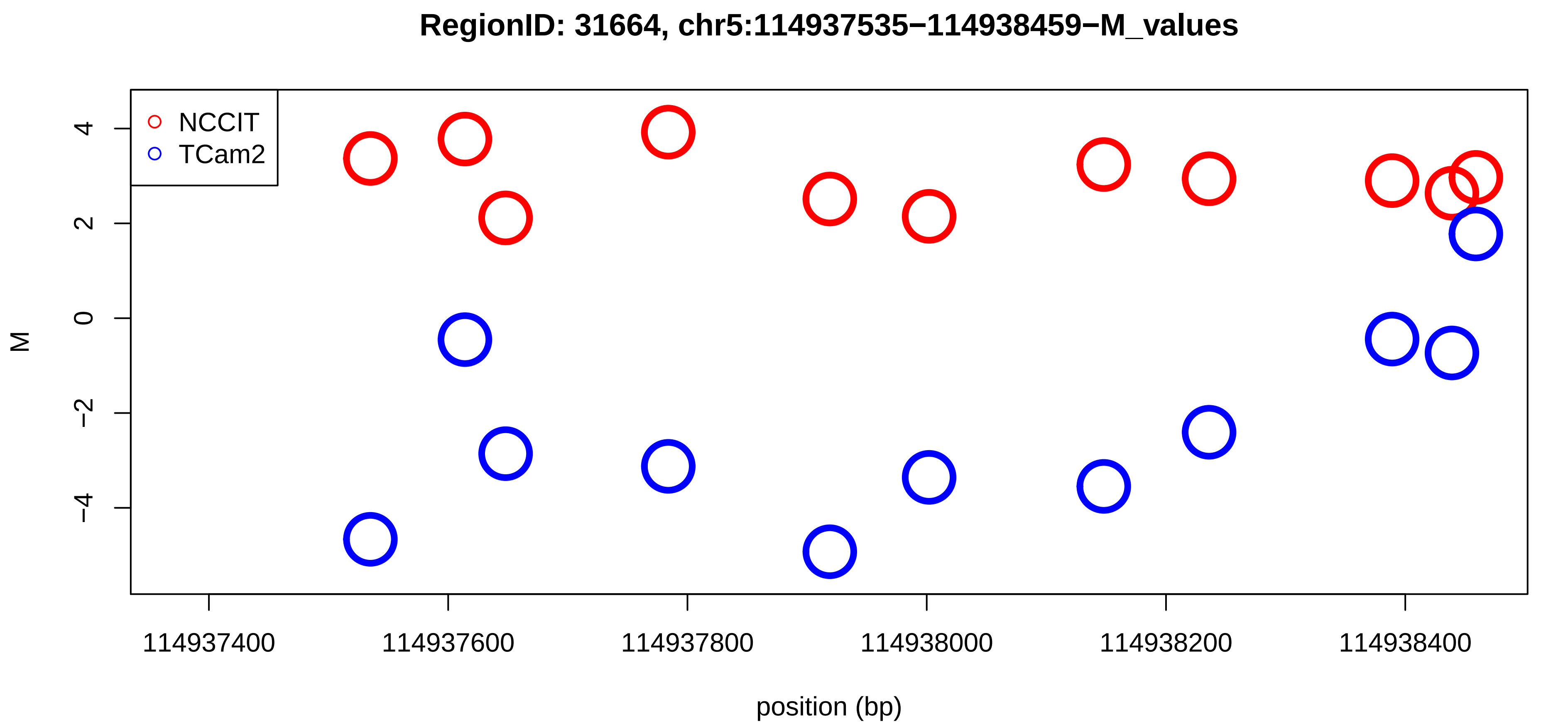
RegionID: 31664, chr5:114937535−114938459−M_values
NCCIT
TCam2
M
4
2
0
−2
−4
114937400
114937600
114937800
114938000
114938200
114938400
position (bp)

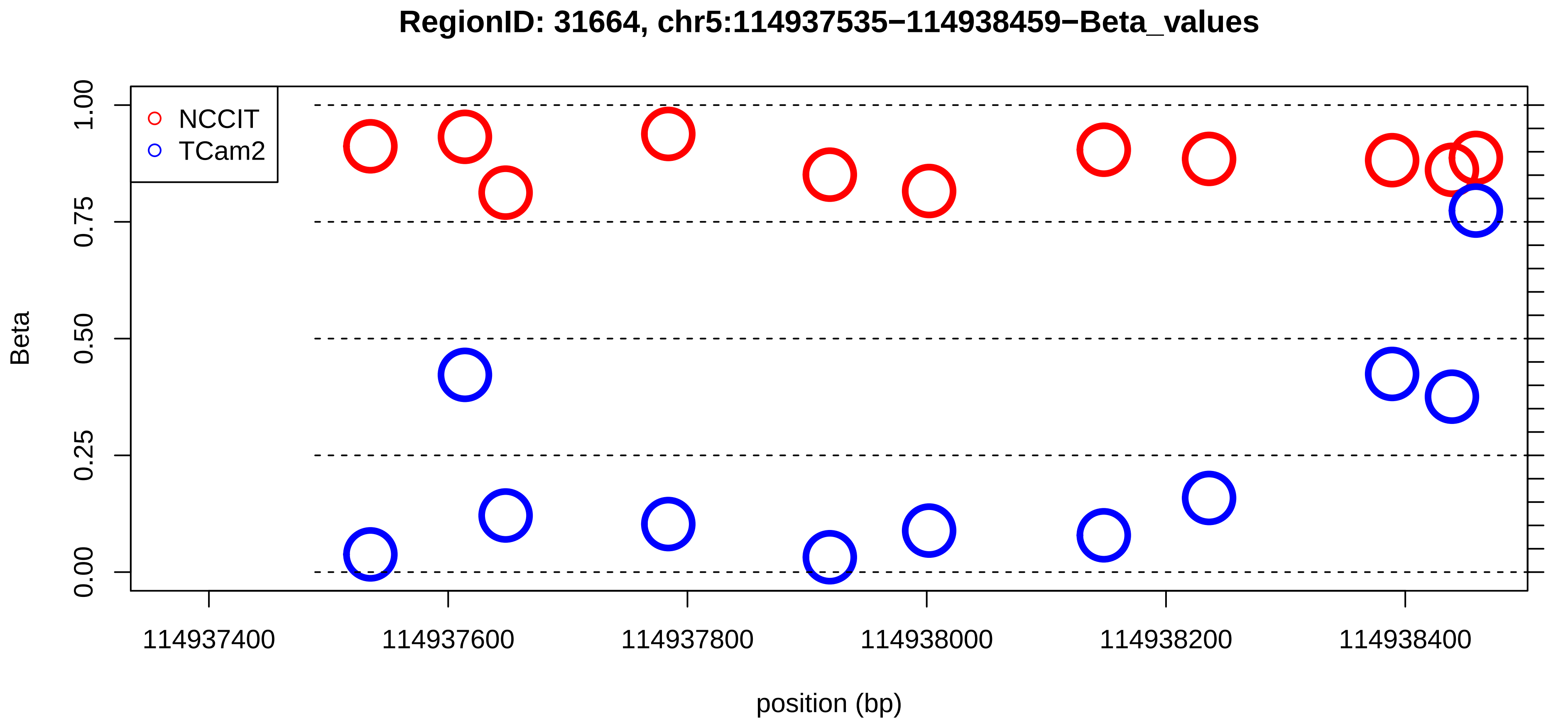
RegionID: 31664, chr5:114937535−114938459−Beta_values
NCCIT
TCam2
Beta
1.00
0.75
0.50
0.25
0.00
114937400
114937600
114937800
114938000
114938200
114938400
position (bp)

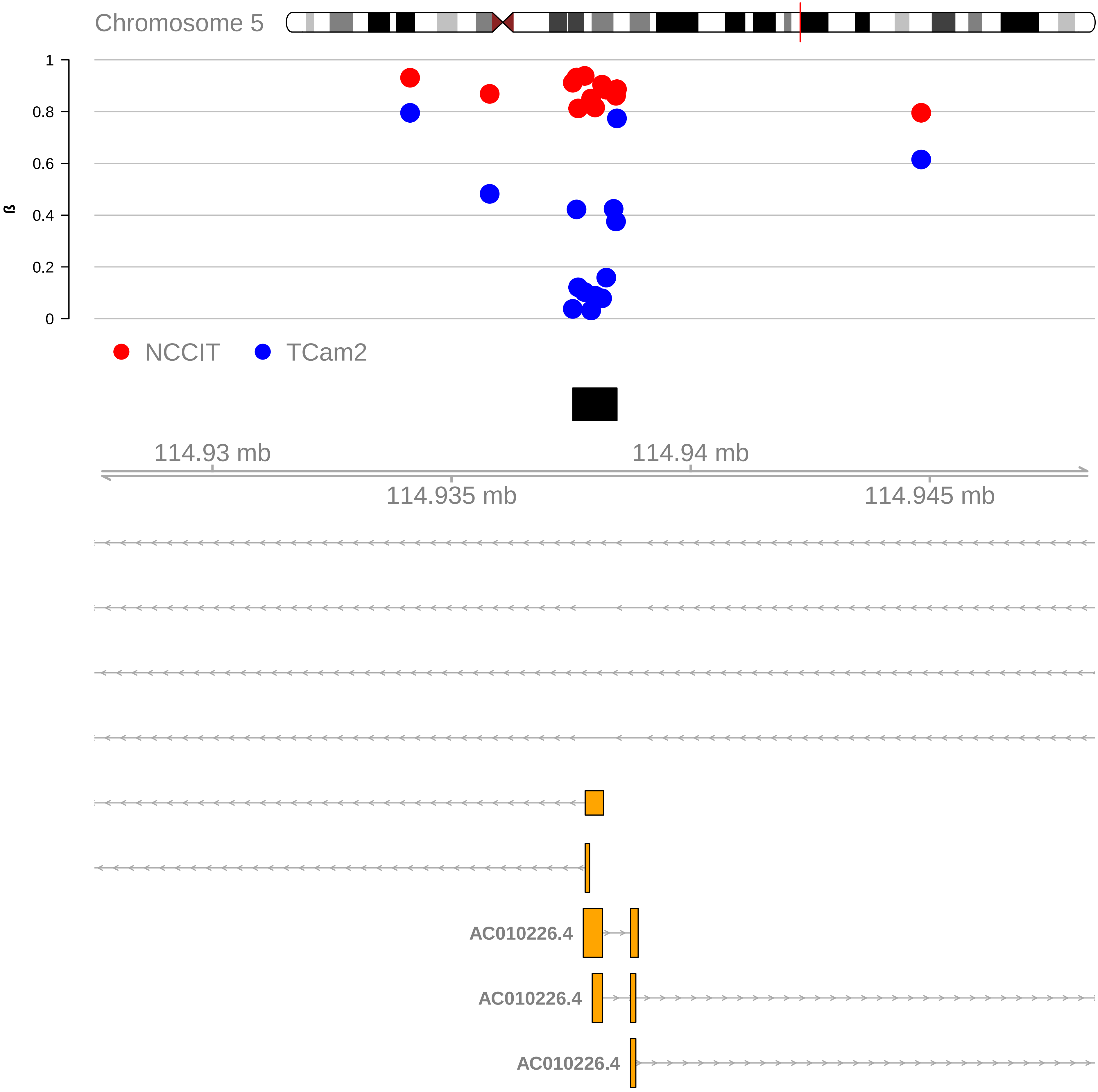

Chromosome 5
1
0.8
0.6
0.4
0.2
0
β
NCCIT
TCam2
114.93 mb
114.94 mb
114.935 mb
114.945 mb
AC010226.4
AC010226.4
AC010226.4
